# Cefiderocol for the Treatment of Infections by VIM-Type-Producing Gram-Negative Bacteria

**DOI:** 10.3390/antibiotics13090874

**Published:** 2024-09-12

**Authors:** Cristina Kirkegaard-Biosca, Ester del Barrio-Tofiño, Miguel Villamarín, Nieves Larrosa, David Campany, Juan José González-López, Ricard Ferrer, Belén Viñado, Laura Doménech, Julia Sellarès-Nadal, Laura Escolà-Vergé, Nuria Fernández-Hidalgo, Ibai Los-Arcos

**Affiliations:** 1 Infectious Diseases Department, Hospital Universitari Vall d’Hebron, 08035 Barcelona, Spain; cristina.kirkegaard@vallhebron.cat (C.K.-B.); miguel.villamarin@vallhebron.cat (M.V.); julia.sellares@vallhebron.cat (J.S.-N.); 2 Department of Medicine, Universitat Autònoma de Barcelona, 08035 Barcelona, Spain; nieves.larrosa@vallhebron.cat (N.L.); juanjo.gonzalez@vallhebron.cat (J.J.G.-L.); ricard.ferrer@vallhebron.cat (R.F.); lescola@santpau.cat (L.E.-V.); 3 Microbiology Department, Hospital Universitari Vall d’Hebron, 08035 Barcelona, Spain; ester.delbarrio@vallhebron.cat (E.d.B.-T.); belen.vinado@vallhebron.cat (B.V.); 4 CIBERINFEC, ISCIII—CIBER de Enfermedades Infecciosas, Instituto de Salud Carlos III, 28029 Madrid, Spain; 5 Pharmacy Department, Hospital Universitari Vall d’Hebron, 08035 Barcelona, Spain; david.campany@vallhebron.cat (D.C.); laura.domenech@vallhebron.cat (L.D.); 6 Critical Care Department, Hospital Universitari Vall d’Hebron, 08035 Barcelona, Spain; 7 Sepsis Organ Dysfunction and Resuscitation (SODIR) Research Group, Vall d’Hebron Institut de Recerca (VHIR), 08035 Barcelona, Spain; 8 Infectious Diseases Unit, Internal Medicine Department, Hospital de la Santa Creu i Sant Pau, 08025 Barcelona, Spain

**Keywords:** metallo-β-lactamase, siderophore, multidrug resistant, extremely drug resistant, carbapenemase

## Abstract

VIM-type-producing Gram-negative bacteria (GNB) infections are difficult to treat. This is a retrospective single-center study of 34 patients who received cefiderocol for the treatment of VIM-type-producing GNB infections, including 25 *Pseudomonas* spp., 7 *Enterobacterales*, and 5 *Achromobacter* sp. Primary outcomes were clinical failure (defined as death, lack of clinical improvement, or a switch to another drug) at day 14 and 30-day all-cause mortality. The median age was 59 years (IQR 53.7–73.4), and the median Charlson comorbidity index was 3.5 (IQR 2–5). The main infections were respiratory tract infections (*n* = 9, 27%) and skin and soft tissue infections (*n* = 9, 27%). Eight patients exhibited bacteremia. In 9/17 patients with a drainable focus, drainage was performed. The median cefiderocol treatment duration was 13 days (IQR 8–24). Five patients (15%) experienced clinical failure on day 14, and the thirty-day mortality rate was 9/34 (27%); two cases occurred because of an uncontrolled infection source, and one was due to a new infection caused by the same bacteria. The other six deaths were unrelated to the index infection. Five patients experienced microbiological recurrence within three months. Susceptibility testing revealed the development of cefiderocol resistance in 1/7 cases with persistent or recurrent positive cultures. Cefiderocol, even in monotherapy, could be considered for the treatment of VIM-type-producing GNB infections.

## 1. Introduction

The treatment of metallo-β-lactamase (MBL)-producing Gram-negative bacteria (GNB) infections is not well defined [1,2]. The Infectious Diseases Society of America (IDSA) 2023 guidelines recommend treatment with ceftazidime–avibactam in combination with aztreonam or cefiderocol monotherapy for infections caused by MBL-producing *Enterobacterales*. For *Pseudomonas aeruginosa*, cefiderocol is recommended, as ceftazidime-avibactam combined with aztreonam offers limited advantage over aztreonam alone, unlike in MBL-producing *Enterobacterales*. [2].

Cefiderocol is a siderophore cephalosporin with excellent in vitro activity against *Pseudomonas aeruginosa*, *Acinetobacter baumannii*, and carbapenem-resistant *Enterobacterales*, including KPC, OXA-48, and MBLs (NDM, IMP, VIM) [3,4]. It consists of a combination of a catechol-type siderophore and a cephalosporin core, utilizing the siderophore–iron complex pathway to penetrate the outer membrane of GNB. This structure and its unique mechanism of action confer enhanced stability against hydrolysis by various β-lactamases. Real-life data on the use of cefiderocol for the treatment of MBL-Gram-negative bacteria (GNB) are emerging [5,6,7,8,9,10]. However, more clinical data are still needed, such as its clinical efficacy in monotherapy or the risk of developing resistance after treatment.

In our center, where VIM (Verona integron-encoded metallo- β-lactamase) is the predominant type of MBL, we started to use cefiderocol to treat VIM-type-producing GNB infections, especially in patients with *P. aeruginosa* infections or severe infections. The aim of this study was to describe the clinical characteristics and outcomes of patients treated with cefiderocol for the treatment of infections caused by VIM-type-producing GNB.

## 2. Methods

We conducted a retrospective study at Vall d’Hebron University Hospital, a 1100-bed tertiary hospital in Barcelona, Spain. The study included all consecutive adult patients (aged ≥18 years) who were diagnosed with an infection caused by VIM-type-producing GNB and treated with cefiderocol for a minimum of 5 days between June 2020 and March 2024. Patients were identified through a pharmacy database, and data were retrospectively extracted from electronic medical records and entered into a specifically designed database. We collected comprehensive data on key epidemiological factors (demographics, comorbidities, immunosuppressive factors), clinical variables (infection type and source control, bacteremia, septic shock, antimicrobial therapy, clinical outcomes), and microbiological characteristics (culture type, microbiological isolation, susceptibility patterns). Patients were followed for at least 90 days after completing antibiotic treatment. During the study period, each patient was prospectively evaluated by an infectious disease physician since the microbiology service reported the results of all multidrug-resistant isolates daily, including VIM-type-producing GNB. Furthermore, the hospital’s Antimicrobial Stewardship Team reviewed prospectively all cefiderocol prescriptions during the study period.

Measures of central tendency and dispersion were used to describe the clinical characteristics of the individuals. Quantitative variables were presented as the mean and standard deviation or median with 25–75 percentiles. For qualitative variables, we used frequencies, absolute, and relative percentages. Statistics were performed with SPSS 23.0 Statistics version 23.

The study protocol received approval from our hospital’s ethics committee (EOM(AG)051/2022(6051)).

### 2.1. Definitions and Outcomes

We defined infections according to the Centers for Disease Control and Prevention criteria [11].

Cefiderocol was administered at a dose of 2 g every 8 h, infused over 3 h. The dosage was adjusted for creatinine clearance according to the manufacturer’s instructions [12]. The infection source control was determined by the research team and defined as the removal of any preexisting contaminated intravascular device or the drainage of intra-abdominal abscesses or other fluid collections thought to be the source of infection [13,14].

Primary outcomes were clinical failure at 14 days and 30-day all-cause mortality from the index culture. Secondary outcomes were infection relapse, microbiological recurrence, and adverse reactions. The presence of diarrhea (whether associated or not with *Clostridioides difficile*), neurotoxicity, or cutaneous reactions was specifically investigated.

Clinical failure was defined as death, the absence of clinical improvement, or the need to switch to another drug due to insufficient response. Infection relapse was defined as the occurrence of a second microbiologically confirmed MBL-producing GNB infection with the reappearance of clinical signs and symptoms at the same infection site. Microbiological recurrence was defined as a new positive culture obtained after completing cefiderocol therapy in patients with available repeated cultures, irrespective of the presence of signs or symptoms of infection. Septic shock was defined as a vasopressor requirement to maintain a mean arterial pressure of ≥65 mm Hg and a serum lactate level greater than 2 mmol/L in the absence of hypovolemia [15].

### 2.2. Microbiological Methods

Bacterial isolates were identified from clinically significant samples by mass spectrometry (Vitek-MS; bioMérieux, Marcy-l’Étoile, France). Cefiderocol susceptibility testing was performed by disk diffusion on unsupplemented Mueller–Hinton agar with cefiderocol 30 μg discs according to EUCAST recommendations [16]. Susceptibility to cefotaxime, ceftazidime/avibactam, aztreonam, imipenem, meropenem, amikacin, ciprofloxacin, trimethoprim–sulfamethoxazole, and colistin was assessed by microdilution using Sensititre TM DKMGN panels (Thermo Fisher, Waltham, MA, USA) according to the manufacturer’s recommendations. Interpretation of the results was performed by applying the EUCAST clinical breakpoints available from 2023 [17]. KPC-type, OXA-48-like, IMP-type, VIM-type, and NDM-type carbapenemase production was confirmed by a lateral flow immunoassay (NG-Test Carba 5 assay; NG-Biotech, ZI Courbouton, France, and the presence of CTX-M-type encoding genes was identified by multiplex real-time PCR (AllplexTM Entero-DR Assay; Seegene, Seoul, Republic of Korea) according to the manufacturer’s instructions.

## 3. Results

**Figure 1:**
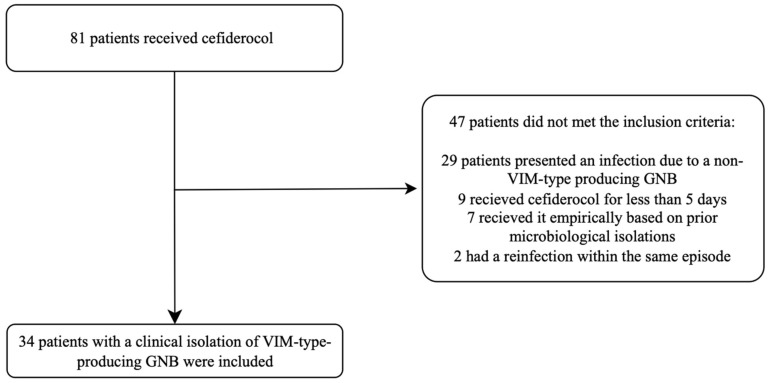
Flow chart representing the patient selection process.

During the study period, 81 patients who received cefiderocol were assessed for eligibility. We included 34 patients with an infection caused by a VIM-type-producing GNB who met the inclusion criteria (Figure 1).

The median age was 59 years (interquartile range [IQR] 53.7–73.4), and 24 patients (71%) were male. The median Charlson comorbidity index was 3.5 (IQR 2–5), and fourteen (41%) patients were solid organ transplant recipients. Nine patients (27%) were in the intensive care unit at the onset of infection. Table 1 summarizes the clinical characteristics and outcomes of the included patients, and Table 2 describes their characteristics in detail.

The most frequent infections were skin and soft tissue infections (*n* = 9, 27%), two of which were extracorporeal membrane oxygenation (ECMO) cannula-related infections with negative blood cultures, followed by respiratory tract infections (*n* = 9, 27%). Eight patients (24%) had positive blood cultures, and one of them presented with septic shock. In 9 out of 17 (53%) patients with a drainable focus, drainage was performed (Table 1). 

Among the 34 patients, 37 VIM-type-producing GNB were isolated as follows: twenty-one *Pseudomonas aeruginosa*, five *Achromobacter* sp., four *Enterobacter cloacae*, four *Pseudomonas putida*, one *Escherichia coli*, one *Raoultella ornithinolytica* and one *Enterobacter hormaechei*. Three patients presented with a coinfection of two different VIM-type-producing GNBs during the same infection episode (Table 3).

Cefiderocol was administered at a standard dose in 20 patients (59%), while 14 patients (41%) were treated with an adjusted dosage due to renal failure. The median duration of cefiderocol treatment was 13 days (IQR 8–24). Cefiderocol was combined with other in vitro-active antibiotics at some point during treatment in nine patients (27%). The antibiotics used in combination were nebulized colistin (*n* = 5), nebulized amikacin (*n* = 1), nebulized aztreonam (*n* = 1), intravenous amikacin (*n* = 1), and intravenous colistin (*n* = 1).

Ceftazidime–avibactam plus aztreonam (CZA+ATM) was not used in any of the patients for different reasons. Among the six patients with *Enterobacterales* infection, ATM administration was not possible for Patients 2, 3, and 19 because ATM was out of stock, for Patient 8 due to neurological toxicity caused by CZA+ATM, or for Patients 9 and 22 due to the concomitant isolation of *P. aeruginosa*. Due to the limited clinical experience in treating *P. aeruginosa* and *Achromobacter* sp. infections with CZA+ATM, the use of cefiderocol was prioritized (Table 1).

Five patients (15%) presented clinical failure on day 14: three due to early deaths unrelated to the infection (Patient 1, 6, and 23) and two patients due to the lack of control of the infectious source (Patients 5 and 10) (Table 1).

The overall 30-day mortality was 9/34 (27%) at a median of 24 (IQR 14–37) days after the initiation of cefiderocol. Four patients died during the course of antimicrobial treatment (4/9; 44%). Two of them had an uncontrolled infection source despite antimicrobial treatment (Patients 5 and 10): one patient had persistent bacteremia due to *Achromobacter* sp. secondary to ECMO cannula-related infection that could not be removed; the other patient, who was a recent allogeneic hematopoietic stem cell transplant recipient, had a tonsillar abscess with an uncontrolled infection in the context of severe neutropenia and disseminated intravascular coagulation. One patient initially treated for a cUTI died due to a new infection of ventilator-associated pneumonia (VAP) caused by the same bacteria (VIM-type-producing *Achromobacter* sp.). The other six patients died from causes unrelated to the index infection according to the clinical assessment of the treating physician: two due to noninfectious respiratory insufficiency, one due to health care-associated pneumonia without microbiological isolation, one due to acute pancreatitis with multiple organ failure, one due to Ritcher’s syndrome with no therapeutic options available, and one due to end-stage renal failure. No adverse effects of cefiderocol were reported.

Among the twenty-five survivors, five (20%) patients experienced microbiological recurrence within 3 months. One patient (Patient 8) had a femoral–femoral bypass graft infection with VIM-type-producing *Enterobacter cloacae*. Initially, due to surgical risk, conservative antibiotic treatment was performed. Despite prolonged antibiotic treatment, the patient experienced relapse of the infection and required a bypass graft explant. The graft cultures were positive for the same bacteria susceptible to cefiderocol, and the infection was successfully treated with a second course of cefiderocol for 4 weeks. Another patient (Patient 18) had asymptomatic bacteriuria and did not receive new antibiotic treatment, whereas the last three patients (Patients 29, 30, and 31) developed a new episode of VAP with the same initial isolation requiring cefiderocol treatment again. 

In eight patients with persistent microbiological isolation or microbiological recurrence after completing treatment, seven were tested for cefiderocol susceptibility. One patient (Patient 5) developed resistance to cefiderocol after 22 days of persistent bacteremia. The other six isolates (Patients 8, 10, 18, 29, 30, and 31) were still susceptible to cefiderocol.

## 4. Discussion

The key findings of our study include a clinical failure rate of 15% and a 30-day mortality of 27% after cefiderocol treatment in patients with VIM-type-producing GNB infections. In other observational studies, the 30-day mortality in patients treated with cefiderocol ranged from 10 to 60% [5,6,7,8,9,10,18]. This is probably due to both the heterogeneity of the underlying disease and the different types of infections. In the largest cohorts of patients treated with cefiderocol for MDR bacteria, a 19% and 37% 30-day mortality rate were reported [6]. In both studies, cefiderocol susceptibility was tested in only about 45% of the isolates. Notably, only 12 [6] and 17 [10] VIM-producing GNB infections were included. Furthermore, in both studies, about half of the patients received combined treatment with other active antibiotics. In our study, only 27% of the cases received other active antibiotics. Most of these treatments involved inhaled antibiotics, resulting in only 6% of patients receiving other active intravenous therapy. 

In our cohort, up to 67% of deaths were not related to the index infection. We believe that the most likely explanation was the severity and high comorbidity of these patients. The increase in clinical failure at 14 days with respect to our previous experience with CZA+ATM for VIM-type-producing GNB [13] was remarkable (15% vs. 8%). This could mainly be due to the lack of an infection source control, which was more frequent with cefiderocol than CZA+ATM (24% vs. 4%). 

The recommended treatments for MBL-producing *Enterobacterales* include CZA+ATM and cefiderocol [2]. In our experience, both treatments are useful against infections caused by VIM-type-producing *Enterobacterales*. The use of CZA+ATM could be chosen in these cases to preserve cefiderocol, although the lack of standardization of the synergistic activity study is a limitation of this therapy. For the treatment of MBL-producing *P. aeruginosa* infections, cefiderocol is currently recommended due to the limited experience of combined treatment and its lower synergistic activity [2]. The experience with cefiderocol in infections caused by *Achromobacter* sp. is very limited. In a series of eight patients with cystic fibrosis treated with cefiderocol, a 92% clinical response was described, but microbiological recurrence occurred in almost all patients [19].

Despite the lack of source control in some cases, we found only one case of resistance development to cefiderocol in our cohort in a patient with 22 days of persistent bacteremia. This resistance could have appeared during treatment or could have been due to undetected cefiderocol heteroresistance, which may have led to the selection of the resistant subpopulation during the course of treatment, as observed by other authors [20]. Resistance to cefiderocol after treatment has been described as similar to other drugs [2]: NDM-type-producing *E. coli* [21], VIM-type-producing *P. aeruginosa* [22], and *K. pneumoniae* [23]. It should be noted that in a similar study carried out in our hospital with ceftolozane–tazobactam for the treatment of infections caused by extensively drug-resistant *P. aeruginosa*, the emergence of resistance was more frequent (4/7 isolates (57%)) [24].

This study has several limitations. First, it was a retrospective and single-center investigation. Furthermore, the sample size was small, there was no control group, and infection types were heterogeneous. However, to the best of our knowledge, this is the largest cohort of VIM-type-producing GNB patients treated with cefiderocol and describes real-world data.

In conclusion, cefiderocol, even in monotherapy, could be considered for the treatment of VIM-type-producing GNB infections.

## Figures and Tables

**Table 1:** Summary of clinical characteristics, antibiotic regimens, and outcome of patients treated with cefiderocol for VIM-type-producing Gram-negative bacteria.

	Patients (*n* = 34)
Men	24 (71)
Age, years, median (IQR)	59 (53.7–73.4)
Age-adjusted Charlson comorbidity index, median (IQR)	3.5 (2–5)
Comorbidities	
Solid organ transplantation	14 (41)
Renal impairment (CrCl < 60 mL/min)	15 (44)
Malignancy	11 (32)
Cardiovascular diseases	7 (21)
Chronic obstructive pulmonary disease	6 (18)
Liver cirrhosis	2 (6)
Hematologic transplantation	1 (3)
Admitted to ICU	9 (27)
Infection type	
Respiratory tract	9 (27)
Skin and soft tissue	9 (27)
Urinary tract	7 (21)
Bone	4 (12)
Bloodstream infection	3 (9)
Intra-abdominal infection	2 (6)
Positive blood cultures	8 (24)
Drainable source	17 (50)
Drainage performed	9 (27)
Days of treatment with cefiderocol, median (IQR)	13 (8–24)
Patients treated with combination therapy	9 (27)
Nebulized antibiotics	7 (21)
Intravenous antibiotics	2 (6)
Clinical failure at 14 days of follow-up	5 (15)
Death	3 (9)
Persistent infection	2 (6)
All-cause mortality at 30 days of follow-up	9 (27)
Microbiological recurrence at 90 days of follow-up among 25 survivors	5 (25)

Data are expressed as the number and percentage unless otherwise indicated.

**Table 2:** Demographic, clinical, and outcome characteristics of the 34 patients with infections caused by VIM-type-producing Gram-negative bacteria treated with cefiderocol.

Patient	Age, yr (Sex)	Underlying Disease	CCI	Type of Infection	Bacterial Species	Bacteremia	Drainable Source/Drainage Performed	Additional Therapy	Days of Treatment	Clinical Failure at Day 14	Mortality at Day 30
1	82 (M)	Renal transplant recipient	7	Primary bacteremia	*P. aeruginosa*	Yes	No	No	8	No	Yes
2	89 (M)	Chronic ischemia right lower limb	14	Chronic osteomyelitis	*E. cloacae*	No	Yes/Yes: Limb amputation	No	13	No	Yes
3	63 (M)	Lung transplant recipient	2	Previous ECMO cannula wound infection	*Raoultella ornithinolytica,* *E. coli*	No	Yes/Yes: Resection of the infected wound	No	13	No	No
4	76 (F)	Diverticulitis	4	Postoperative deep wound infection	*Achromobacter* sp., *P. aeruginosa*	No	Yes/No: Undrained abscess	No	8	No	No
5	56 (M)	COVID-19 pneumonia	3	Primary bacteremia and ECMO cannula infection	*Achromobacter* sp.	Yes	Yes/No: ECMO cannula not retired	Intravenous colistin (after 22 days of cefiderocol)	27	Yes	Yes
6	39 (M)	COVID-19 pneumonia in a lung transplant recipient	1	ECMO cannula wound infection + tracheobronchitis	*P. aeruginosa*	No	Yes/No: ECMO cannula not retired	Nebulized colistin	6	Yes	Yes
7	51 (M)	Acute pancreatitis	2	Catheter-associated urinary infection	*Achromobacter* sp.	No	No	No	11	No	Yes
8	78 (F)	Renal transplant recipient	10	Femoro-femoral bypass graft infection	*E. cloacae*	No	Yes/No: By-pass not explanted	No	13	No	No
9	65 (M)	Renal transplant recipient	6	Obstructive pyelonephritis	*E. cloacae,* *P. aeruginosa*	Yes	Yes/Yes: Ureteral stent placement	No	27	No	No
10	59 (M)	Acute myeloid leukemia and recent allo-HSCT	3	Tonsillar abscess	*P. aeruginosa*	Yes	Yes/No: Undrained abscess	Intravenous amikacin (during the first 3 days)	22	Yes	Yes
11	41 (M)	NK/T-cell lymphoma	2	Infected pancreatic necrosis	*P. aeruginosa*	No	Yes/Yes: Abscess drainage	No	62	No	No
12	56 (F)	Follicular lymphoma with persistent COVID-19	2	Catheter-associated UTI	*Achromobacter* sp.	No	No	No	5	No	No
13	67 (M)	Lung transplant recipient	2	Acute tracheobronchitis	*P. aeruginosa*	No	No	Nebulized colistin	7	No	No
14	77 (F)	Acute myeloid leukemia	5	Soft tissue infection	*P. aeruginosa*	No	No	No	14	No	No
15	61 (M)	Hemorrhagic shock	7	Catheter-associated UTI	*Achromobacter* sp.	No	No	No	7	No	Yes
16	56 (M)	Tibial osteomyelitis	0	Chronic osteomyelitis	*P. putida*	No	Yes/Yes: Osteomyelitis debridement	No	14	No	No
17	70 (M)	COPD	5	Nosocomial pneumonia	*P. aeruginosa*	Yes	No	No	25	No	Yes
18	51 (M)	Renal transplant recipient	3	Renal graft pyelonephritis	*P. aeruginosa*	No	No	No	17	No	No
19	54 (M)	Elbow osteomyelitis	1	Osteosynthesis-associated infection	*E. cloacae*	No	Yes/Yes: Osteosynthesis implant removal	No	32	No	No
20	58 (M)	Renal transplant recipient	4	Postoperative deep wound infection	*P. aeruginosa*	No	Yes/No: Undrained abscess	No	28	No	No
21	67 (M)	Lung transplant recipient	4	Acute tracheobronchitis	*P. aeruginosa*	Yes	No	Nebulized colistin	15	No	No
22	54 (F)	Lung transplant recipient	3	Acute tracheobronchitis	*Enterobacter hormaechei*	No	No	Nebulized amikacin	6	No	No
23	45 (M)	Richter’s Syndrome	2	Intestinal bacterial translocation	*P. aeruginosa*	Yes	No	No	9	No	No
24	53 (F)	Open tibia fracture	1	Tibial osteomyelitis	*P. aeruginosa*	No	Yes/Yes: Osteomyelitis debridement	No	38	No	Yes
25	54 (F)	Metastatic kidney cancer	5	Catheter-related bloodstream infection	*P. aeruginosa*	Yes	Yes/Yes: Catheter removal	No	5	No	No
26	78 (F)	Renal transplant recipient	9	Postoperative deep wound infection	*P. putida*	No	Yes/Yes: Abscess drainage	No	41	No	No
27	44 (F)	Renal transplant recipient	3	Postoperative deep wound infection	*P. putida*	No	Yes/No: Undrained abscess	No	12	No	No
28	49 (M)	Lung transplant recipient	4	Acute tracheobronchitis	*P. aeruginosa*	No	No	No	14	No	No
29	75 (M)	Status epilepticus	3	Ventilator-associated pneumonia	*P. aeruginosa*	No	No	Nebulized colistin	9	No	No
30	77 (M)	Multiple organ dysfunction syndrome	8	Acute tracheobronchitis	*P. aeruginosa*	No	No	No	10	No	No
31	59 (F)	Pulmonary fibrosis	1	Acute tracheobronchitis	*P. aeruginosa*	No	No	Nebulized colistin	8	No	No
32	80 (M)	Meige syndrome (tracheostomy)	4	Acute tracheobronchitis	*P. aeruginosa*	No	No	Nebulized aztreonam	10	No	No
33	56 (M)	Acute lymphoblastic leukemia	4	Prostatic abscess	*P. aeruginosa*	No	Yes/No: Undrained abscess	No	33	No	No
34	63 (M)	Renal transplant recipient	5	Renal graft pyelonephritis	*P. putida*	No	No	No	18	No	No

Abbreviations: allo-HSCT: allogeneic hematopoietic stem cell transplantation; CCI, Charlson comorbidity index; ECMO, extracorporeal membrane oxygenation; F, female; M, male; UTI, urinary tract infection; and COPD, chronic obstructive pulmonary disease.

**Table 3:** In vitro antibiotic susceptibility pattern of 37 isolates from 34 patients with VIM-type-producing Gram-negative bacteria infections treated with cefiderocol.

Patient	Bacterial Species	Acquired Beta-Lactamases	MIC (mg/L) (S/I/R)									FDC Zone Diameter (mm)	FDC Susceptibility in Persistent or Recurrent Isolates
			MEM	IPM	ATM	CZA	C/T	CIP	AMK	CST	SXT		
1	*P. aeruginosa*	VIM	>16 (R)	>16 (R)	8 (I)	>16 (R)	>32 (R)	>2 (R)	8 (S)	1 (S)	NAp	22 (S)	-
2	*E. cloacae*	VIM+CTX-M	4 (I)	4 (I)	≤0.5 (S)	>16 (R)	>32 (R)	1 (R)	≤4 (S)	0.5 (S)	>8 (R)	24 (S)	-
3	*Raoultella ornithinolytica*	VIM	1 (S)	4 (S)	≤0.5 (S)	>16 (R)	>32 (R)	≤0.06 (S)	≤4 (S)	0.5 (S)	>8 (R)	25 (S)	-
	*E. coli*	VIM	1 (S)	2 (S)	≤0.5 (S)	>16 (R)	>32 (R)	≤0.06 (S)	≤4 (S)	≤0.25 (S)	>8 (R)	24 (S)	-
4	*P. aeruginosa*	VIM	>16 (R)	>16 (R)	8 (I)	>16 (R)	>32 (R)	1 (R)	8 (S)	1 (S)	NAp	32 (S)	-
	*Achromobacter* sp.	VIM	NA	NA	NA	NA	NA	NA	NA	NA	NA	NA	-
5	*Achromobacter* sp.	VIM	>16 (R)	>16	>32	>16	>32	>2	>32	4	>8 (R)	33 (S)	10 (R)
6	*P. aeruginosa*	VIM	>16 (R)	>16 (R)	8 (I)	>16 (R)	>32 (R)	1 (R)	≤4 (S)	1 (S)	NAp	23 (S)	-
7	*Achromobacter* sp.	VIM	16 (R)	>16	32	>16	>32	2	>32	4	>8 (R)	28 (S)	-
8	*E. cloacae*	VIM+CTX-M	4 (I)	4 (I)	≤0.5 (S)	>16 (R)	>32 (R)	2 (R)	≤4 (S)	0.5 (S)	>8 (R)	22 (S)	22 (S)
9	*E. cloacae*	VIM+CTX-M	8 (I)	4 (I)	>32 (R)	>16 (R)	>32 (R)	1 (R)	≤4 (S)	0.5 (S)	>8 (R)	22 (S)	-
	*P. aeruginosa*	VIM	>16 (R)	>16 (R)	16 (I)	>16 (R)	>32 (R)	>2 (R)	≤4 (S)	1 (S)	NAp	22 (S)	-
10	*P. aeruginosa*	VIM	>16 (R)	>16 (R)	32 (R)	>16 (R)	>32 (R)	>2 (R)	≤4 (S)	1 (S)	NAp	25 (S)	25 (S)
11	*P. aeruginosa*	VIM	NA	NA	NA	NA	NA	NA	NA	NA	NA	NA	-
12	*Achromobacter* sp.	VIM	>16 (R)	>16	>32	>16	>32	2	>32	4	>8 (R)	22 (S)	-
13	*P. aeruginosa*	VIM	NA	NA	NA	NA	NA	NA	NA	NA	NA	NA	-
14	*P. aeruginosa*	VIM	>16 (R)	>16 (R)	4 (I)	>16 (R)	>32 (R)	2 (R)	≤4 (S)	1 (S)	NAp	26 (S)	-
15	*Achromobacter* sp.	VIM	NA	NA	NA	NA	NA	NA	NA	NA	NA	NA	NA
16	*P. putida*	VIM	>16 (R)	16 (R)	8 (I)	>16 (R)	>32 (R)	>2 (R)	4 (S)	1 (S)	NAp	28 (S)	-
17	*P. aeruginosa*	VIM	>16 (R)	>16 (R)	8 (I)	>16 (R)	>32 (R)	>2 (R)	8 (S)	1 (S)	NAp	24 (S)	-
18	*P. aeruginosa*	VIM	>16 (R)	>16 (R)	32 (R)	>16 (R)	>32 (R)	>2 (R)	≤4 (S)	1 (S)	NAp	23 (S)	23 (S)
19	*E. cloacae*	VIM+CTX-M	1 (S)	4 (I)	≤0.5 (S)	>16 (R)	>32 (R)	1 (R)	≤4 (S)	0.5 (S)	>8 (R)	27 (S)	-
20	*P. aeruginosa*	VIM	>16 (R)	>16 (R)	8 (I)	>16 (R)	>32 (R)	>2 (R)	8 (S)	2 (S)	NAp	26 (S)	-
21	*P. aeruginosa*	VIM	>16 (R)	>16 (R)	8 (I)	>16 (R)	>32 (R)	1 (R)	≤4 (S)	0.5 (S)	NAp	31 (S)	-
22	*Enterobacter hormaechei*	VIM+KPC	8 (I)	8 (R)	32 (R)	>16 (R)	>32 (R)	>2 (R)	≤4 (S)	1 (S)	>8 (R)	22 (S)	-
23	*P. aeruginosa*	VIM	8 (I)	16 (R)	32 (R)	>16 (R)	>32 (R)	>2 (R)	>32 (R)	1 (S)	NAp	25 (S)	-
24	*P. aeruginosa*	VIM	>16 (R)	>16 (R)	>32 (R)	>16 (R)	>32 (R)	>2 (R)	≤4 (S)	1 (S)	NAp	25 (S)	-
25	*P. aeruginosa*	VIM	>16 (R)	>16 (R)	8 (I)	>16 (R)	>32 (R)	>2 (R)	8 (S)	2 (S)	NAp	28 (S)	-
26	*P. putida*	VIM	>16 (R)	>16 (R)	32 (R)	>16 (R)	>32 (R)	2 (R)	≤4 (S)	1 (S)	NAp	24 (S)	-
27	*P. putida*	VIM	>16 (R)	>16 (R)	8 (I)	>16 (R)	>32 (R)	>2 (R)	8 (S)	2 (S)	NAp	24 (S)	-
28	*P. aeruginosa*	VIM	>16 (R)	>16 (R)	16 (I)	>16 (R)	>32 (R)	>2 (R)	≤4 (S)	2 (S)	NAp	23 (S)	-
29	*P. aeruginosa*	VIM	>16 (R)	16 (R)	32 (R)	>16 (R)	>32 (R)	0,12 (I)	≤4 (S)	2 (S)	NAp	26 (S)	26 (S)
30	*P. aeruginosa*	VIM	>16 (R)	16 (R)	1 (I)	>16 (R)	>32 (R)	0,06 (I)	≤4 (S)	1 (S)	NAp	29 (S)	25 (S)
31	*P. aeruginosa*	VIM	>16 (R)	>16 (R)	4 (I)	>16 (R)	>32 (R)	>2 (R)	8 (S)	2 (S)	NAp	32 (S)	28 (S)
32	*P. aeruginosa*	VIM	>16 (R)	>16 (R)	32 (R)	>16 (R)	>32 (R)	>2 (R)	≤4 (S)	0.5 (S)	NAp	27 (S)	-
33	*P. aeruginosa*	VIM	>16 (R)	>16 (R)	8 (I)	>16 (R)	>32 (R)	>2 (R)	8 (S)	2 (S)	NAp	24 (S)	-
34	*P. putida*	VIM	>16 (R)	>16 (R)	8 (I)	>16 (R)	>32 (R)	>2 (R)	8 (S)	2 (S)	NAp	23 (S)	-
Total susceptibility (%) *			24	20	70	0	0	13	97	100	0	100	86

AMK: amikacin; ATM: aztreonam; CIP: ciprofloxacin; CST: colistin; CTX: cefotaxime; CZA: ceftazidime/avibactam; C/T: ceftolozane–tazbactam; I: susceptible increased exposure; IPM: imipenem; MEM: meropenem; NA: not available; NAp: not applicable; R: resistant; S: susceptible; and SXT: trimethoprim–sulfamethoxazole. * Because EUCAST only has clinical breakpoints for meropenem and cotrimoxazole regarding *Achromobacter* sp., the MICs of other antibiotics have not been categorized.

## Data Availability

The datasets presented in this article are not readily available because we do not have permission from the ethical commitee.
