# Correction: Kirkegaard-Biosca et al. Cefiderocol for the Treatment of Infections by VIM-Type-Producing Gram-Negative Bacteria. *Antibiotics* 2024, *13*, 874

**DOI:** 10.3390/antibiotics14010004

**Published:** 2024-12-24

**Authors:** Cristina Kirkegaard-Biosca, Ester del Barrio-Tofiño, Miguel Villamarín, Nieves Larrosa, David Campany, Juan José González-López, Ricard Ferrer, Belén Viñado, Laura Doménech, Julia Sellarès-Nadal, Laura Escolà-Vergé, Nuria Fernández-Hidalgo, Ibai Los-Arcos

**Affiliations:** 1 Infectious Diseases Department, Hospital Universitari Vall d’Hebron, 08035 Barcelona, Spain; cristina.kirkegaard@vallhebron.cat (C.K.-B.); miguel.villamarin@vallhebron.cat (M.V.);; 2 Department of Medicine, Universitat Autònoma de Barcelona, 08035 Barcelona, Spain; nieves.larrosa@vallhebron.cat (N.L.); juanjo.gonzalez@vallhebron.cat (J.J.G.-L.); ricard.ferrer@vallhebron.cat (R.F.); lescola@santpau.cat (L.E.-V.); 3 Microbiology Department, Hospital Universitari Vall d’Hebron, 08035 Barcelona, Spain; ester.delbarrio@vallhebron.cat (E.d.B.-T.); belen.vinado@vallhebron.cat (B.V.); 4 CIBERINFEC, ISCIII—CIBER de Enfermedades Infecciosas, Instituto de Salud Carlos III, 28029 Madrid, Spain; 5 Pharmacy Department, Hospital Universitari Vall d’Hebron, 08035 Barcelona, Spain; david.campany@vallhebron.cat (D.C.); laura.domenech@vallhebron.cat (L.D.); 6 Critical Care Department, Hospital Universitari Vall d’Hebron, 08035 Barcelona, Spain; 7 Sepsis Organ Dysfunction and Resuscitation (SODIR) Research Group, Vall d’Hebron Institut de Recerca (VHIR), 08035 Barcelona, Spain; 8 Infectious Diseases Unit, Internal Medicine Department, Hospital de la Santa Creu i Sant Pau, 08025 Barcelona, Spain

**Error in Table 2**

In the original publication [1], there was a mistake in Table 2, published as follows: Patients 1 and 23 presented clinical failure at day 14, as reported in the text. In addition, by error patient 23 should also appear as mortality at day 30: Yes, and patient 24 mortality at day 30 should be No.

The corrected version of Table 2 appears below. The authors state that the scientific conclusions are unaffected. This correction was approved by the Academic Editor. The original publication has also been updated.

**Table 2:** Demographic, clinical, and outcome characteristics of the 34 patients with infections caused by VIM-type-producing Gram-negative bacteria treated with cefiderocol.

Patient	Age, yr (Sex)	Underlying Disease	CCI	Type of Infection	Bacterial Species	Bacteremia	Drainable Source/Drainage Performed	Additional Therapy	Days of Treatment	Clinical Failure at Day 14	Mortality at Day 30
1	82 (M)	Renal transplant recipient	7	Primary bacteremia	*P. aeruginosa*	Yes	No	No	8	Yes	Yes
2	89 (M)	Chronic ischemia right lower limb	14	Chronic osteomyelitis	*E. cloacae*	No	Yes/Yes: Limb amputation	No	13	No	Yes
3	63 (M)	Lung transplant recipient	2	Previous ECMO cannula wound infection	*Raoultella ornithinolytica*, *E. coli*	No	Yes/Yes: Resection of the infected wound	No	13	No	No
4	76 (F)	Diverticulitis	4	Postoperative deep wound infection	*Achromobacter* sp., *P. aeruginosa*	No	Yes/No: Undrained abscess	No	8	No	No
5	56 (M)	COVID-19 pneumonia	3	Primary bacteremia and ECMO cannula infection	*Achromobacter* sp.	Yes	Yes/No: ECMO cannula not retired	Intravenous colistin (after 22 days of cefiderocol)	27	Yes	Yes
6	39 (M)	COVID-19 pneumonia in a lung transplant recipient	1	ECMO cannula wound infection + tracheobronchitis	*P. aeruginosa*	No	Yes/No: ECMO cannula not retired	Nebulized colistin	6	Yes	Yes
7	51 (M)	Acute pancreatitis	2	Catheter-associated urinary infection	*Achromobacter* sp.	No	No	No	11	No	Yes
8	78 (F)	Renal transplant recipient	10	Femoro-femoral bypass graft infection	*E. cloacae*	No	Yes/No: By-pass not explanted	No	13	No	No
9	65 (M)	Renal transplant recipient	6	Obstructive pyelonephritis	*E. cloacae*, *P. aeruginosa*	Yes	Yes/Yes: Ureteral stent placement	No	27	No	No
10	59 (M)	Acute myeloid leukemia and recent allo-HSCT	3	Tonsillar abscess	*P. aeruginosa*	Yes	Yes/No: Undrained abscess	Intravenous amikacin (during the first 3 days)	22	Yes	Yes
11	41 (M)	NK/T-cell lymphoma	2	Infected pancreatic necrosis	*P. aeruginosa*	No	Yes/Yes: Abscess drainage	No	62	No	No
12	56 (F)	Follicular lymphoma with persistent COVID-19	2	Catheter-associated UTI	*Achromobacter* sp.	No	No	No	5	No	No
13	67 (M)	Lung transplant recipient	2	Acute tracheobronchitis	*P. aeruginosa*	No	No	Nebulized colistin	7	No	No
14	77 (F)	Acute myeloid leukemia	5	Soft tissue infection	*P. aeruginosa*	No	No	No	14	No	No
15	61 (M)	Hemorrhagic shock	7	Catheter-associated UTI	*Achromobacter* sp.	No	No	No	7	No	Yes
16	56 (M)	Tibial osteomyelitis	0	Chronic osteomyelitis	*P. putida*	No	Yes/Yes: Osteomyelitis debridement	No	14	No	No
17	70 (M)	COPD	5	Nosocomial pneumonia	*P. aeruginosa*	Yes	No	No	25	No	Yes
18	51 (M)	Renal transplant recipient	3	Renal graft pyelonephritis	*P. aeruginosa*	No	No	No	17	No	No
19	54 (M)	Elbow osteomyelitis	1	Osteosynthesis-associated infection	*E. cloacae*	No	Yes/Yes: Osteosynthesis implant removal	No	32	No	No
20	58 (M)	Renal transplant recipient	4	Postoperative deep wound infection	*P. aeruginosa*	No	Yes/No: Undrained abscess	No	28	No	No
21	67 (M)	Lung transplant recipient	4	Acute tracheobronchitis	*P. aeruginosa*	Yes	No	Nebulized colistin	15	No	No
22	54 (F)	Lung transplant recipient	3	Acute tracheobronchitis	*Enterobacter hormaechei*	No	No	Nebulized amikacin	6	No	No
23	45 (M)	Richter’s Syndrome	2	Intestinal bacterial translocation	*P. aeruginosa*	Yes	No	No	9	Yes	Yes
24	53 (F)	Open tibia fracture	1	Tibial osteomyelitis	*P. aeruginosa*	No	Yes/Yes: Osteomyelitis debridement	No	38	No	No
25	54 (F)	Metastatic kidney cancer	5	Catheter-related bloodstream infection	*P. aeruginosa*	Yes	Yes/Yes: Catheter removal	No	5	No	No
26	78 (F)	Renal transplant recipient	9	Postoperative deep wound infection	*P. putida*	No	Yes/Yes: Abscess drainage	No	41	No	No
27	44 (F)	Renal transplant recipient	3	Postoperative deep wound infection	*P. putida*	No	Yes/No: Undrained abscess	No	12	No	No
28	49 (M)	Lung transplant recipient	4	Acute tracheobronchitis	*P. aeruginosa*	No	No	No	14	No	No
29	75 (M)	Status epilepticus	3	Ventilator-associated pneumonia	*P. aeruginosa*	No	No	Nebulized colistin	9	No	No
30	77 (M)	Multiple organ dysfunction syndrome	8	Acute tracheobronchitis	*P. aeruginosa*	No	No	No	10	No	No
31	59 (F)	Pulmonary fibrosis	1	Acute tracheobronchitis	*P. aeruginosa*	No	No	Nebulized colistin	8	No	No
32	80 (M)	Meige syndrome (tracheostomy)	4	Acute tracheobronchitis	*P. aeruginosa*	No	No	Nebulized aztreonam	10	No	No
33	56 (M)	Acute lymphoblastic leukemia	4	Prostatic abscess	*P. aeruginosa*	No	Yes/No: Undrained abscess	No	33	No	No
34	63 (M)	Renal transplant recipient	5	Renal graft pyelonephritis	*P. putida*	No	No	No	18	No	No

Abbreviations: allo-HSCT, allogeneic hematopoietic stem cell transplantation; CCI, Charlson comorbidity index; ECMO, extracorporeal membrane oxygenation; F, female; M, male; UTI, urinary tract infection; and COPD, chronic obstructive pulmonary disease.
